# Versatile and Simple Approach to Determine Astrocyte Territories in Mouse Neocortex and Hippocampus

**DOI:** 10.1371/journal.pone.0069143

**Published:** 2013-07-23

**Authors:** Antje Grosche, Jens Grosche, Mark Tackenberg, Dorit Scheller, Gwendolyn Gerstner, Annett Gumprecht, Thomas Pannicke, Petra G. Hirrlinger, Ulrika Wilhelmsson, Kerstin Hüttmann, Wolfgang Härtig, Christian Steinhäuser, Milos Pekny, Andreas Reichenbach

**Affiliations:** 1 Paul Flechsig Institute of Brain Research, Faculty of Medicine, University of Leipzig, Leipzig, Germany; 2 Institute of Human Genetics, Faculty of Medicine, University of Regensburg, Regensburg, Germany; 3 Center for Brain Repair and Rehabilitation, Department of Clinical Neuroscience and Rehabilitation, Institute of Neuroscience and Physiology, Sahlgrenska Academy, University of Gothenburg, Gothenburg, Sweden; 4 Institute of Cellular Neurosciences, Medical Faculty, University of Bonn, Bonn, Germany; University of Kentucky, United States of America

## Abstract

**Background:**

Besides their neuronal support functions, astrocytes are active partners in neuronal information processing. The typical territorial structure of astrocytes (the volume of neuropil occupied by a single astrocyte) is pivotal for many aspects of glia–neuron interactions.

**Methods:**

Individual astrocyte territorial volumes are measured by Golgi impregnation, and astrocyte densities are determined by S100β immunolabeling. These data are compared with results from conventionally applied methods such as dye filling and determination of the density of astrocyte networks by biocytin loading. Finally, we implemented our new approach to investigate age-related changes in astrocyte territories in the cortex and hippocampus of 5- and 21-month-old mice.

**Results:**

The data obtained by our simplified approach based on Golgi impregnation were compared to previously published dye filling experiments, and yielded remarkably comparable results regarding astrocyte territorial volumes. Moreover, we found that almost all coupled astrocytes (as indicated by biocytin loading) were immunopositive for S100β. A first application of this new experimental approach gives insight in age-dependent changes in astrocyte territorial volumes. They increased with age, while cell densities remained stable. In 5-month-old mice, the overlap factor was close to 1, revealing little or no interdigitation of astrocyte territories. However, in 21-month-old mice, the overlap factor was more than 2, suggesting that processes of adjacent astrocytes interdigitate.

**Conclusion:**

Here we verified the usability of a simple, versatile method for assessing astrocyte territories and the overlap factor between adjacent territories. Second, we found that there is an age-related increase in territorial volumes of astrocytes that leads to loss of the strict organization in non-overlapping territories. Future studies should elucidate the physiological relevance of this adaptive reaction of astrocytes in the aging brain and the methods presented in this study might be a powerful tool to do so.

## Introduction

Protoplasmic astrocytes are the primary glial cell subpopulation in the brain gray matter. Besides maintaining topographic relationships and structural integrity, these cells contribute to ion and transmitter homeostasis [1], metabolic support, control of neuronal energy supply, detoxification of reactive oxygen species [2]–[4], and control of neuronal activity [5], [6]. To carry out these diverse tasks, astrocytes rely on their elaborate morphology. Their processes are strikingly complex, with numerous arborizations and ramifications that enwrap neuronal structures and blood vessels and thereby constitute an anatomical link between these two compartments. Astroglial cells and their processes form co-existing domains, such as nano-, micro-, and macrodomains, which probably interact in distinct ways with single synaptic elements, groups of functionally related synapses, and even large functional assemblies of neurons, respectively [7], [8]. For instance, repetitive side branches of Bergmann glial cell stem processes in the cerebellum (glial microdomains) each appear to interact with a small group of synapses on a Purkinje cell dendrite [9]. At a larger scale, neurons in the barrel cortex of mice apparently interact primarily with astrocytes within their barrel, as a glial macrodomain [10]. Thus, a glial domain corresponds to a territory of neuropil that is penetrated by, and interacts with, an astrocyte process, an astrocyte, or even a (coupled) network of astrocytes [7].

This raises the question of how the spatial arrangement of astrocytes is organized with respect to their neighbors. If every astrocyte occupies a defined volume of the neuropil, does this mean that no other astrocyte invades the same volume, or is there an overlap between the territories occupied by neighboring astrocytes? An overlap would ensure support of a given neural tissue compartment even when an astrocyte becomes dysfunctional. However, astrocytes avoid intense interdigitation with processes from neighboring cells by “tiling” – a process that results in territorial volume overlap of only 4–6% between adjacent astrocytes in adult mouse hippocampus [11]–[13]. In the cerebellum and other murine brain regions, astroglial territories overlap considerably. There, each microcompartment of the neuropil is penetrated by processes of two Bergmann astroglial cells [9], [14]. Earlier morphometric studies of rat cerebral cortex by electron microscopy suggested an overlap factor of about 3 [15], [16].

Unfortunately, the data are sparse and were obtained from different brain regions, by different methods, from animals of different age, and even from different species, making comparisons difficult. The sparseness of the data is unsurprising, as intracellular dye filling of neighboring astrocytes and morphometric electron microscopy studies are sophisticated, time-consuming methods.

To obtain information on the spatial arrangement of astrocytes in different brain regions requires reliable data on the number of astrocytes and the volume they access. Ogata and Kosaka [13] assessed data on projection areas of astrocytes in Golgi-impregnated hippocampal slices. The calculated territorial volume of astrocytes closely matched results from intracellular dye labeling on fixed slices. Thus, Golgi impregnation can be used to estimate the astrocyte territorial volume.

We devised a simple, versatile method to assess the overlap between adjacent astrocyte territories. Specifically, the average volume accessed by a single astrocyte is measured on stacks of confocal images of Golgi-labeled astrocytes and multiplied by the number of astrocytes in a larger unit volume, counted on histological sections immunolabeled for an astrocyte-specific protein, S100β. This “total astrocyte-accessed volume” is divided by the unit brain volume. In the case of astrocyte tiling, the resulting overlap factor should be close to 1. Values higher than 1 would indicate sharing of territories between adjacent astrocytes, and values below 1 would indicate incomplete penetration of the whole neuropil by astrocyte processes.

Using this method, we compared the organization of astrocyte territories in the hippocampus and cerebral cortex of mice at 5 months and 21 months of age. These territories might differ in the brains of adult and old mice, because astrocytes are thought to become hypertrophic with advancing age and because neurons in these two regions appear to be especially prone to age-related degeneration [17]–[19]. Moreover, reactive gliosis has been proposed to affect to age-related pathologies such as Alzheimer's disease and amyotrophic lateral sclerosis [20]–[24]. Finally, the intermediate filament protein glial fibrillary acidic protein (GFAP) is upregulated and GFAP-positive astrocyte processes become hypertrophic during “physiological” aging of the brain or in disease situations, and this might have an effect on astrocyte territories or their organization [25]–[29].

## Materials and Methods

### Animals

Female and male C57Bl6, 129Ola and 129Sv mice were used in the study. All experiments were performed in accordance with the European Communities Council Directive 86/609/EEC and German guidelines for welfare of experimental animals (Tierschutzgesetz) and were approved by the local authorities (Landesdirektion Sachsen, permit numbers T18/12A and T09/13).

### Golgi procedure

All mice were perfused with 4% phosphate-buffered paraformaldehyde and decapitated, and the brains were removed. From each mouse, one hemisphere was processed with the Golgi method as described [30] with minor modifications. In brief, 6-mm-thick blocks were postfixed in 4% phosphate-buffered paraformaldehyde containing 8% glutaraldehyde for 2–4 d. The tissue was then quickly rinsed with distilled water, processed with 2.5% and 3.5% potassium bichromate for 1 d each, treated with 25% silver nitrate for 2 d, and embedded in 16% celloidin. Subsequently, 150-µm-thick slices were cut with a microtome and embedded in Canada balsam in xylene.

### Immunohistochemical staining

The other brain hemisphere of each mouse was postfixed in 4% paraformaldehyde, equilibrated with 30% phosphate-buffered sucrose, and cut into 30-µm-thick coronal frozen sections with a freezing microtome. For immunofluorescence labeling of the astroglial marker S100β, free-floating sections were incubated overnight with a rabbit anti-S100β antibody (1 ∶400; Swant, Bellinzona, Switzerland). The tissue was then incubated with carbocyanine (Cy) 2–conjugated donkey anti-rabbit IgG (20 µg/ml; Dianova, Hamburg, Germany) for 1 h. Next, the remaining free binding sites of Cy2-anti-rabbit IgG were blocked with 50% rabbit antiserum for 3 h. The sections were then incubated overnight with digoxigenylated rabbit anti-GFAP antibodies (10 µg/ml) as described [31]. GFAP immunoreactivity was revealed by incubation for 1 h with Cy3-anti-digoxin (20 µg/ml; Dianova, Hamburg, Germany).

In control experiments, omission of primary antibodies resulted in the expected absence of cellular labeling. In addition, Cy3-anti-GFAP antibodies produced the same staining pattern as digoxigenylated anti-GFAP antibodies successively revealed by Cy3-anti-digoxin (data not shown).

### Determining astrocyte territorial volume

**Figure 1:**
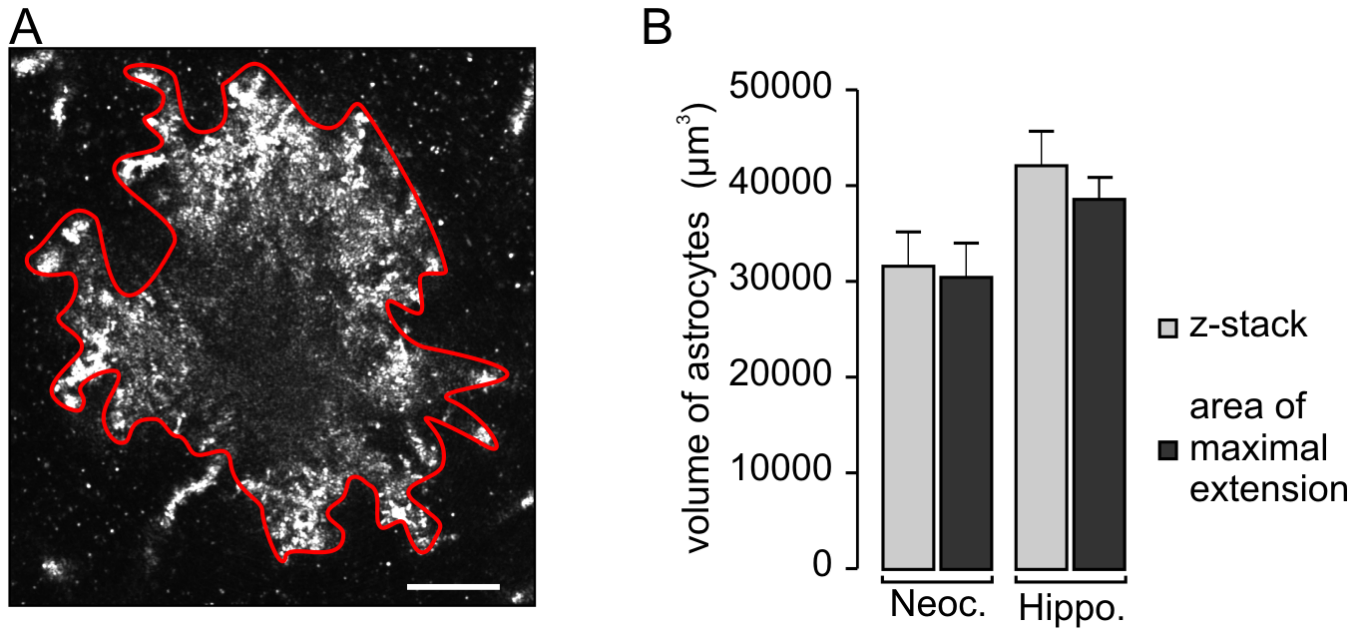
Comparison of two methods to assess astrocyte territorial volume. For description of the z-stack method, see the Methods chapter of the text. (**A**) The area of maximal extension of a hippocampal astrocyte was recorded on a Golgi-impregnated brain slice from an adult mouse using the reflection mode. The approximate borders of an astrocyte were encircled only considering clearly attached compartments (red line), and the enclosed area was calculated using the LSM software and was treated as that of a virtual circle, representing the central section of a virtual sphere. Next, the radius of this virtual circle could be calculated. This was taken to get the volume of the astrocyte territory. .Scale bar, 10 µm. (**B**) Two methods were compared by determining volumes of astrocyte territories on the same slices from adult mice (n = 4) for both cortex and hippocampus. Although astrocyte volume tended to be slightly underestimated when the calculation was based on the area of maximal extension, no significant difference was found. Each bar represents the values from 20–48 cells. Neoc., neocortex; Hippo., hippocampus.

We focused on two brain regions: the neocortex (primary and secondary somatosensory cortex, layers II–IV) and the CA3 area (stratum moleculare) of the hippocampus. The volume accessed by a single astrocyte (i.e., by its territory) was defined as the space over which the astrocyte processes extend, including the finest elaborations of the main branches. To calculate this volume, three-dimensional z-stacks from Golgi-impregnated brain slices were obtained with a Zeiss confocal laser-scanning microscope 510 Meta in reflection mode. Single cells were split into 1-µm-thick optical slices (approximately 40–70 slices per astrocyte, depending on its diameter). The area of each cross-section was measured with Zeiss Image Analyzer Software. With this technique, multiple two-dimensional areas per slice could be calculated up to the cell's three-dimensional volume. Alternatively, the area of maximal extension of each astrocyte was recorded on maximum projections of an astrocyte. This area was treated as circular and used to calculate the volume of a theoretical “astrocyte globe” (Fig. 1A). Finally, all the values were corrected by a shrinkage factor. This was necessary because the Golgi staining data were combined with the immunohistochemical staining data, and the influence of the different embedding techniques had to be ruled out.

To calculate this factor, defined as the degree of tissue shrinkage after embedding, the thickness of the 20 embedded slices (included preparations from adult and aged mice) was measured and normalized to the thickness of the original slices (150 µm). Subtraction from 100% results in shrinkage of about 13.8%. Assuming that all structures shrink to about the same degree, we included this factor in our calculations, which led to slightly increased astrocyte volumes. Potential shrinkage from postfixation was not determined and might lead to a slight underestimation of the volume of astrocyte territories. Shrinkage associated with embedding was not considered for the immunohistochemical staining used to determine the astrocyte cell number, as we could count all astrocytes in the 30-µm-thick slice (frozen sections).

### Data analysis

#### Morphometric analysis of cell process architecture

To assess the complexity of the process architecture, we counted the branchings of all main processes up to 15 µm from the soma center of each astrocyte.

#### Counting astrocytes

Immunohistochemically stained sections were analyzed for the number of cells expressing the calcium-binding protein S100β, a reliable marker of astrocytes in mammalian brain [32]. Using an Achroplan 20×/0.5 W Ph2 objective (Zeiss, Oberkochen, Germany), we estimated the number of astrocytes per unit volume for each studied brain region. The pinhole was set to obtain 5.5-µm-thick optical sections, and small z-stacks (average, five optical sections) were recorded to ensure that all cells throughout the thickness of the slice were imaged. Five representative stacks (460.7 µm ×460.7 µm) from each brain area were scanned per mouse. Finally, we calculated the number of astrocytes per mm^3^ as the counted number of astrocytes/[area where astrocytes were counted (mm^2^) x slice thickness (mm)].

#### Calculating the overlap factor

Next, we calculated the overlap factor for multiple astrocytes within the observed volume of brain tissue by multiplying the mean astrocyte territorial volume by the number of cells (estimated by counting S100β-positive cells), which showed us the whole three-dimensional space occupied by all astrocytes summed up. This number, divided by the volume of the measured brain tissue, results in the overlap factor, which indicates the extent to which cell processes of individual astrocytes overlap with each other.

## Results

### Determination of astrocyte territorial volume based on golgi staining

We applied Golgi-impregnation on murine brain slices and used a confocal microscope in reflection mode to examine the cells (Fig. 1A). The Golgi technique delineates virtually every fine detail of a cell [33], enabling us to accurately define the volume of astrocyte territories. This volume can be determined in two ways: by reconstruction from z-stack recordings and by calculation based on the area of maximal extension of each cell (Fig. 1A). If the territory is assumed to be spherically symmetrical, the latter method allows efficient analysis of an ample number of cells. Thus, before starting the main study, we determined whether both methods yield comparably accurate results. We calculated the territorial volume from z-stacks recorded from each astrocyte (Fig. 1A). In the second approach, we assumed that each astrocyte occupies a more or less spherical area. If so, it should be possible to measure the area of the maximal extension of an astrocyte, to re-calculate this area as a circle, and calculate the volume as a virtual globe. We used adult mice to assess data with both methods (n = 15 cells for each method). Indeed, both methods yielded well-fitting results (Fig. 1B).

**Figure 2:**
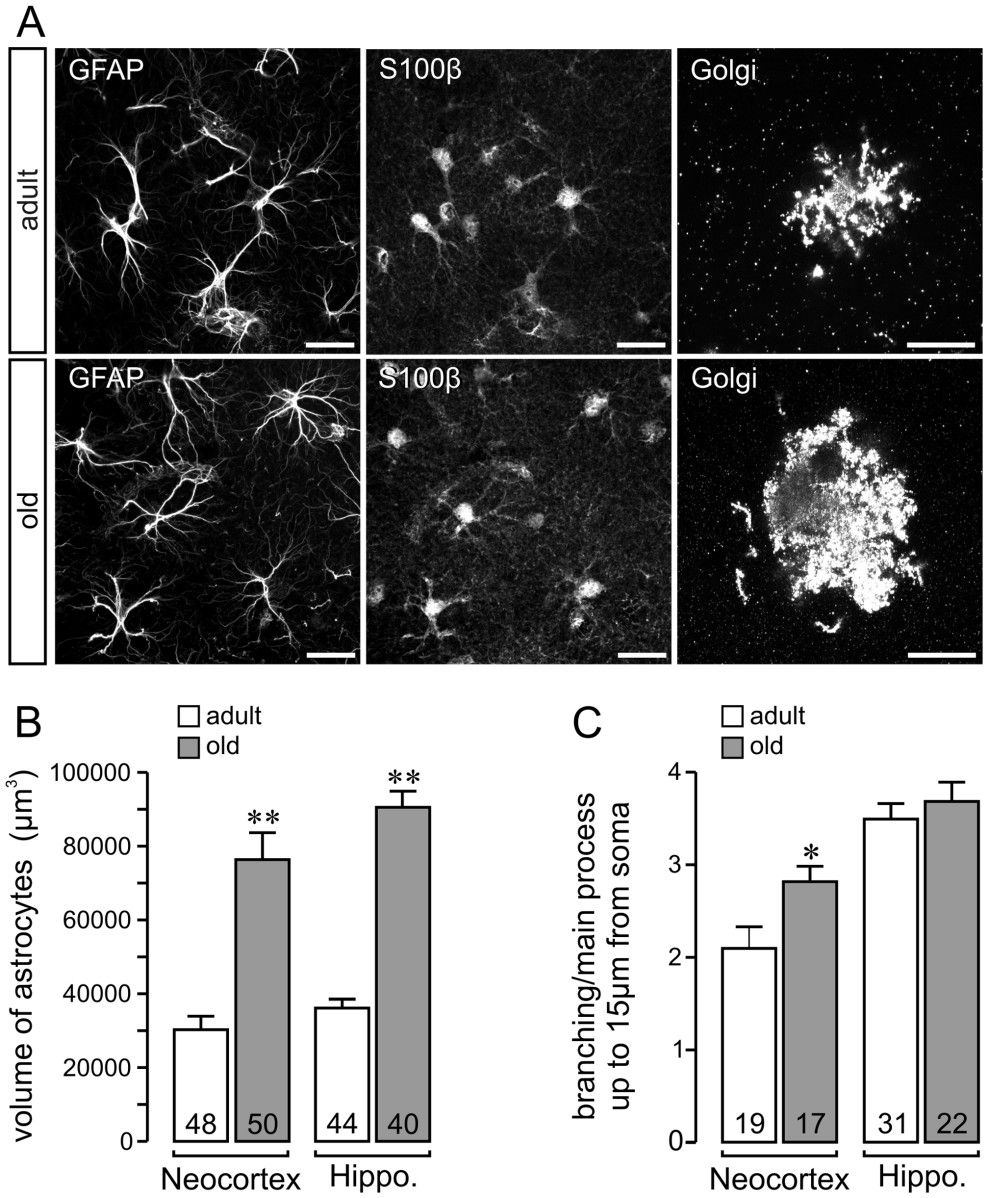
Morphometric analysis of astrocytes in the neocortex and in the hippocampus. (**A**) Representative scans from hippocampal astrocytes (stratum moleculare). Astrocytes were revealed by GFAP and S100β immunolabeling or by Golgi impregnation. Scale bars, 20 µm. (**B**) Volume of astrocyte territories calculated from the area of maximal extension in Golgi-impregnated brain slices. (**C**) The number of branchings per main process within 15 µm from the cell soma was determined. *P<0.05, **P<0.01 vs.values of adult mice. Each column includes cells from 3–5 mice. Absolute cell numbers are given in each column.

Additionally, we compared the results of the present study with data from earlier dye filling experiments performed on adult mice ([29]; Table 1). The observed tendency of hippocampal astrocytes penetrating a larger volume of neuropil than neocortical astrocytes is reflected in data sets obtained by dye filling experiments or basing on the Golgi impregnation technique indicating that both methods yield similarly exact results. Finally, we decided to proceed with the simplified approach using the maximal extension, which allowed a rapid analysis of a notable cell number (Fig. 2B).

**Table 1:** Comparison of astrocyte territorial volumes from adult mice determined on the basis of Golgi impregnation using the calculation from the area of maximal extension with data previously obtained from dye filling experiments [29].

Area	Technique	Territory volume (*10^3^ µm^3^±SEM)	n cells	n mice
Neocortex				
	Golgi impreg.	31±4	49	4 (5 months old)
	dye filling	26±3	18	5 (10 months old)
Hippocampus				
	Golgi impreg.	38±3	44	4 (5 months old)
	dye filling	44±1	45	7 (5 months old)

### Morphometric analysis and determination of astrocyte territorial volume

To test our method, we determined the effect of aging on astrocytes in the cortex and hippocampus in 5- and 21-months-old mice. Since astrocyte architecture in mice is considered to be mature at the age of 5 months [34], we refer to the 5-months-old mice as “adult” and to the 21-months-old mice as “old”. Figure 2A shows representative micrographs from the cell labeling, which was the basis for the morphometric analysis. First, we determined the astrocyte territories calculated from the maximal extension of the cells in Golgi-impregnated brain slices. The mean volume of an astrocyte territory was 30,541±3593 µm^3^ in the neocortex and 38,361±3174 µm^3^ in the hippocampus of adult mice (Fig. 2B). Territorial volumes of neocortical astrocytes were larger in old mice (76,555±7285 µm^3^) than in adult mice. Territorial volumes in hippocampal astrocytes were also more than twofold larger in old mice (93,570±3856 vs. 38,361±3174 µm^3^) (Fig. 2B). Thus, age is an important determinant of astrocyte territorial volume. Moreover, in cortical astrocytes, but not hippocampal astrocytes, we found an age-dependent increase in the number of bifurcations on S100β-positive main astrocyte cell processes (Fig. 2C).

### Astrocyte density in the neocortex and hippocampus

**Figure 3:**
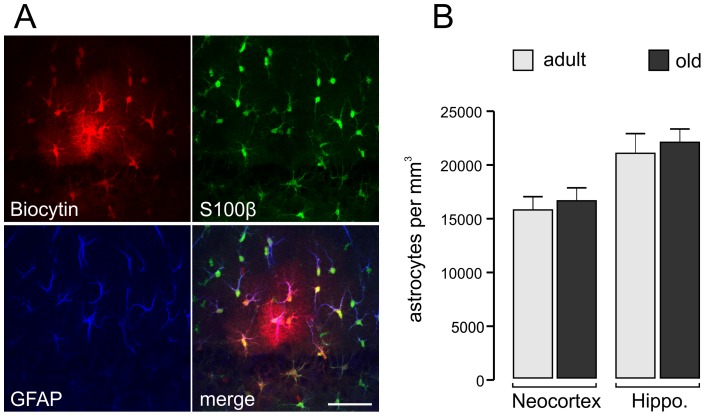
Astrocyte cell densities in hippocampal and cortical brain slices. (**A**) Verification of S100β as suitable astrocyte marker to quantify cell densities. An individual astrocyte was filled with biocytin to reveal coupling within the network of protoplasmic astrocytes in the hippocampus. The same slice was labeled for S100β and GFAP. Scale bar, 50 µm. (**B**) The number of astrocytes stays constant between 5 and 21 months of age.

We also determined the number of astrocytes per volume of brain tissue to calculate the overlap factor. It has been proposed earlier that S100β may be used as a reliable marker for the entire population of astrocytes [32], [35]. Since we were interested in “classical” protoplasmic astrocytes, which are intensely coupled by gap junctions [36], we carried out the following experiment to verify this assumption. Vital hippocampal slices of adult mice were perfused on the stage of a microscope, and an electrophysiologically characterized protoplasmic astrocyte (glial cell with glutamate uptake currents that are mediated by two distinct glutamate transporters) was injected with biocytin (Fig. 3A). After the dye had spread into the coupled astrocyte network, fluorescent cells per unit volume were counted. Thereafter, the sections were fixed and immunolabeled for GFAP and S100β, and immunolabeled cells within the same unit volume were counted. Only a subpopulation of dye-coupled astrocytes was labeled with antibodies against GFAP (213 of 359 coupled cells, 59%); the vast majority were labeled with antibodies against S100β (Fig. 3A). A few dye-coupled cells showed no S100β label, but almost the same number of cells was S100β -positive but not coupled (Table 2). We therefore used S100β immunohistochemistry to count astrocytes in cortical and hippocampal brain slices. The number of astrocytes per mm^3^ did not change with age in cortical or hippocampal slices (15,696±860 in adult cortex and 16,490±872 in old cortex; 20,904±1924 in adult hippocampus and 22,047±1350 in old hippocampus; n = 4 animals each) (Fig. 3B).

**Table 2:** Validation of S100β immunolabeling to count actrocytes in hippocampal brain slices.

	Coupled	S100β	Coupled/S100β	Coupled/No S100β	Not coupled/S100β
number of cells	537	561	451	86	110
% of coupled cells			84.0	16.0	
% of S100β cells			80.4		19.6

### Overlap factor

**Figure 4:**
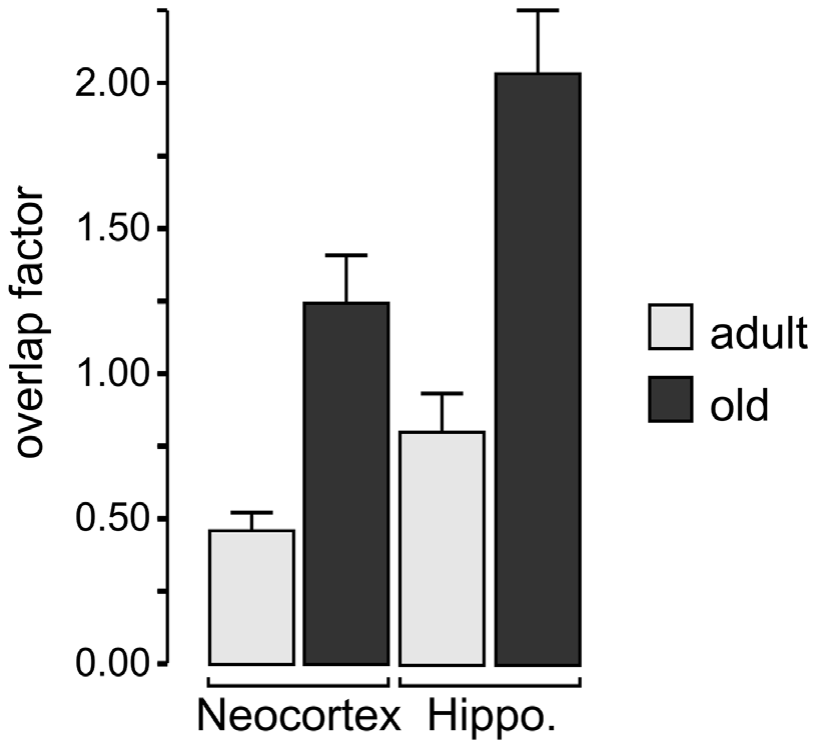
Overlap factor determined from neocortical and hippocampal brain slices.

Finally, to estimate the extent of interdigitation between processes of neighboring astrocytes, we calculated the overlap factor multplying the absolute number of astrocytes by the average astrocyte territorial volume and divided the result by the volume of the brain tissue studied. In adult mice, the resulting overlap factor was 0.47 in the cortex and 0.86 in the hippocampus (Fig. 4). In old mice, however, the overlap factor was 1.26 in the cortex and 2.08 in the hippocampus. Thus, the overlap factor more than doubled between 5 and 21 months of age.

## Discussion

In this study, we established a fast, reliable method to quantitatively assess the morphology and spatial arrangement of astrocytes in different brain regions. Using this method, we showed that aging correlates with remarkable increases in astrocyte cell volume and in the overlap of astrocyte territories. The tiling of astrocyte territories led to a negligible overlap of individual astrocyte territories in adult brain, whereas both the size and overlap of the territories increased in old brains. This increase may be required to compensate for increased metabolic demands and decreasing functional capabilities of aged astrocytes.

### A simple method to characterize the spatial arrangement of astrocytes

Previous work in this field focused on analyzing defined groups of astrocytes filled with different fluorescent dyes [11], [13], [37] or genetically labeled by expression of fluorescent proteins [12], [38], [39]. Both approaches allow exact analysis of the intimate interaction of a limited number of cells. However, both are dependent on GFAP expression, which excludes a subpopulation of astrocytes [40], [41], and dye-filling of cells requires an intense manual effort. Further, extrapolation of these data to astrocytes in larger brain regions has to be made with caution. It is unknown whether the dye is evenly distributed and reaches all the finest processes and spongiform elaborations of protoplasmic astrocytes. Moreover, since astrocytes differ in size, morphology, and gene expression [38], [40], [42]–[46], genetic approaches are limited by the availability of promoters driving the expression of reporter proteins in specific cell types. For example, mice expressing enhanced green fluorescent protein under the control of the GFAP promoter might be an imperfect tool to determine the morphology of astrocytes in healthy cortex, as only a subpopulation of cortical astrocytes expresses GFAP [40]. Here, the recently generated Cx43 knock-in ECFP mouse should be advantageous [38].

Our approach, based on Golgi staining and S100β immunohistochemistry, will make it possible to acquire comprehensive data sets from astrocytes largely independent of the brain region and of the heterogeneity of the astrocyte population since we found that data from dye filling experiments on astrocytes of adult mice [29] were comparable to results obtained in our present study and since S100β is an abundantly expressed marker of astrocytes [40], [47]. In the adult hippocampus and cortex, some NG2 cells also express S100β [43], [48]. However, our data (Fig. 3A, Table 2) reveal that this error is counterbalanced by the occurrence of some “true” (i.e., dye-coupled) astrocytes that fail to express detectable S100β. Thus, it seems reasonable to assume that both astrocyte cell densities and average territory sizes can be reliably assessed by the methods used in our study.

Nonetheless, for both brain regions studied the resulting overlap factors in our study are significantly lower than 1.0 as had been assessed by earlier dye-filling experiments [29]. These have led to the conclusion that astrocytes form a tight network of discrete territories which touch each other [11], [12], [49]. This discrepancy might be explained by a basic methodological difference. In the dye-filling experiments, two (or several) adjacent astrocytes were dye-injected. This can only be done in such areas of the neuropil where neither large blood vessels nor big neuronal cell bodies are present which would decompose the astrocyte network. However in mice, the number of neurons exceeds that of non-neuronal cells (including astrocytes) by a factor of 3 [50] and many of their somata have similar volumes as the territories of astrocytes. Furthermore, some space is occupied by blood vessels (about 1% of cortical volume [50] and other non-neuronal cells, and is not available for astrocyte territories. Taken together, these ‘blocked’ spaces may cause lower overlap factors assessed in average brain volumes as compared to overlap factors obtained in small tissue compartments where the astrocyte territories can touch each other. Nevertheless, our method may be a simple, highly useful alternative to other approaches; it delivers, at least, reasonable lower limit estimates of the average overlap factors. As the general problem should be very similar for given brain regions of adult and old mice, the method can certainly be applied to reveal changes in the territorial organization of astrocytes during aging.

### The astrocyte territorial volume and overlap factor increase with age

Considerable work has been done to characterize the spatial organization of protoplasmic astrocytes in brain gray matter. For example, the reported values of the average volume occupied by a hippocampal astrocyte, varies from 16,400 µm^3^ [51] to 65,900 µm^3^ [11] in rats and from 38,400 µm^3^ (present study), to 43,400 µm^3^ [29] and 85,300 µm^3^ [13] in mice. These differences presumably reflect differences in the methods used and in the ages and genetic backgrounds of the animals. Despite these differences in the estimated volume accessed by single astrocytes, a consensus developed regarding the overlap between adjacent astrocyte territories. By a process called tiling, astrocytes limit the overlap of their processes, thereby creating a patchwork of exclusive astrocyte territories [11]–[13].

We largely confirmed these data, as the overlap factor we calculated that was less than, but close to 1 in adult mice, both in the hippocampus and in the neocortex. Importantly, we found that age is a primary determinant of the mean astrocyte territorial volume in both regions. The territory infiltrated by the fine processes of an average astrocyte more than doubled between 5 and 21 months. Yet, the astrocytes showed few signs of reactive gliosis, such as a slight increase in the extension of their main processes (indicated by the increased size of their territories) and their enhanced branching. These findings do not necessarily contradict earlier studies consistently demonstrating an age-related upregulation of GFAP in astrocytes in general, and, specifically, in the hippocampus [52], [53], as GFAP-labeling only depicts the main processes but not the complete astrocyte morphology. Notably, the cell density remained rather constant whereas the overlap factor of astrocyte territories increased from less than 1 to over 2, indicating that astrocyte territories do not overlap in adult mice but overlap considerably in old mice.

Since astrocytes are strictly organized into a network of non-overlapping territories in adult mice, it is tempting to speculate that this is the morphological equivalent of the functional units that glial cells build with the neuronal structures they enwrap [11], [12], [45] – a hypothesis that remains to be confirmed. Others suggested that the intricate glia-to-neuron interaction is confined to glial microdomains [9] or to the dense meshwork of perisynaptic astrocyte processes in a defined spatial arrangement of synapses rather than to the mere morphological boundaries of an astrocyte [8], [54].

It is less easy to explain the increase in the astrocyte territorial volume and, consequently, in the astrocyte overlap in old mice. Reactive astrocytes in murine epileptic brain show similar structural changes, including a 10-fold increase in the overlap factor [49]. These astrocytes displayed impaired clearance of extracellular K+, which was associated with neuronal hyperexcitability [55]–[59]. Interestingly, valproate, a common antiepileptic drug, suppressed the seizures and reduced the overlap of astrocyte processes [49].

It is not known whether the reorganization of astrocyte territories in epilepsy is the reason for or a consequence of neuronal hyperexcitability. In traumatic brain injury, both the astrocyte territories and the limited overlap of neighboring astrocyte territories are unchanged [29]. Generally, aging is associated with only comparably mild signs of astrocyte activation and reactive gliosis [26], [52], [53], [60], [61]. Therefore, it is not very likely that the changes we observed are caused by any specific “pathology of aging”. Rather, aging may activate some beneficial responses of astrocytes, such as counteracting oxidative stress causally linked to many age-related changes in the brain [3], [62]–[64]. Astrocytes are the key defenders against oxidative stress in the brain [2], [65], and the antioxidant protection they provide is fully active even in senescent mice [3]. So, if one assumes that the antioxidant capacity of an astrocyte in the old brain is sustained at a level similar to that of the adult, and that oxidative stress increases due to accumulation of reactive oxygen species such as H_2_O_2_ [3], [61], an increasing overlap of astrocyte territories might facilitate effective clearance of this surplus of oxidative stress. Of course, this hypothesis needs to be tested. The formation of an extensive network of Golgi stacks is associated with advancing age in astrocytes [25], which points to enhanced metabolic activity of astrocytes in the aged brain. Interestingly, a substantial reduction in the percentage of brain tissue occupied by astrocytes has been linked to bipolar disorders and schizophrenia in humans [66].

Taken together, we present a versatile, simple method to assess the volume of astrocyte territories and to determine astrocyte cell densities in various brain regions. First results obtained with this approach underline the importance to analyze animals of exactly known age if one wants to obtain information about whether and how, in addition to age, pathophysiological mechanisms affect the size and overlap of astrocyte territories.
